# Title: Is Celiac Trunk Revascularization Necessary After High-Flow Pancreaticoduodenal Arterial Arcades Aneurysm Retrograde Embolization?

**DOI:** 10.3390/jcm13237063

**Published:** 2024-11-22

**Authors:** Mohamed Salim Jazzar, Hicham Kobeiter, Mario Ghosn, Raphael Amar, Youssef Zaarour, Athena Galletto Pregliasco, Pascal Desgranges, Vania Tacher, Mostafa El Hajjam, Haytham Derbel

**Affiliations:** 1 Medical Imaging Department, Henri Mondor University Hospital Assistance Publique-Hôpitaux de Paris, 51 Avenue du Maréchal de Lattre de Tassigny, 94010 Créteil, France; 2 Faculty of Health Sciences, University of Paris Est-Creteil, 94000 Créteil, France; 3 Institut Mondor de Recherche Biomédicale, Inserm U955, Team 8, 94000 Créteil, France; 4 Medical Imaging Department, Ambroise Paré University Hospital Assistance Publique-Hôpitaux de Paris, 9 Av. Charles de Gaulle, 92100 Boulogne-Billancourt, France; 5 Vascular Surgery Department, Henri Mondor University Hospital Assistance Publique-Hôpitaux de Paris, 51 Avenue du Maréchal de Lattre de Tassigny, 94010 Créteil, France; 6 Institut Mondor de Recherche Biomédicale, Inserm U955, Team 18, 94000 Créteil, France; 7 Faculty of Medicine, University of Paris Saclay, 91190 Gif-sur-Yvette, France

**Keywords:** MeSH terms, mesenteric artery, superior, radiology, interventional, aneurysm, ruptured, hemorrhage, angiography

## Abstract

**Background and Objective:** High-flow pancreaticoduodenal artery (PDA) aneurysms secondary to celiac trunk occlusion or stenosis have a high risk of rupture. Embolization offers a less invasive alternative to surgery. We evaluated the effectiveness and safety of retrograde embolization via the superior mesenteric artery of high-flow PDA aneurysms without celiac trunk revascularization. **Methods:** This retrospective bicentric study included patients who underwent embolization of high-flow PDA aneurysms due to significant celiac trunk stenosis or occlusion. All patients underwent pre-interventional dynamic contrast-enhanced computed tomography. Retrograde embolization was performed using microcoils and/or liquid agents without celiac trunk revascularization. Follow up involved clinical and radiological assessment at one month. Technical and clinical success were evaluated, and complications were categorized as minor or major. **Results:** Twenty-three patients (mean age 65 ± 14 years; 52% male) were included. Emergency embolization was required in 12 patients (52%). The technical success rate was 100%. Patients were monitored for a median of 16 months. Clinical success was 87%. No hemorrhagic recurrences were observed. Minor complications occurred in two cases. One major complication involved splenic infarction due to glue migration, requiring splenectomy and intensive unit care admission. **Conclusions:** Retrograde embolization of high-flow PDA aneurysms is effective and safe without needing celiac trunk revascularization.

## 1. Introduction

Aneurysms of the pancreaticoduodenal arcades (PDAs) are uncommon and are estimated to be less than 2% among all visceral artery aneurysms. However, they could be life-threatening if left untreated [1]. Evidence suggests that, unlike other visceral aneurysms, the risk of PDA aneurysm rupture is not significantly affected by age, aneurysm size, number of aneurysms, or underlying cause. Rupture rates for untreated aneurysms can be as high as 50%, and reported mortality rates following rupture range from 20% to 50%, underscoring the critical importance of comprehensive identification and treatment of PDA aneurysms [2,3,4,5,6].

The pathophysiology of these aneurysms often involves the compensatory hypertrophy of the pancreaticoduodenal arteries secondary to celiac trunk occlusion or significant stenosis, commonly caused by median arcuate ligament syndrome, which affects approximately 4% of the general population, but also more rarely by atherosclerosis or inflammatory diseases [1]. In such cases, the increased blood flow and pressure through collateral pathways lead to heightened arterial wall stress and aneurysm formation [7,8,9,10].

The treatment of PDA aneurysms lacks consensus, particularly between surgical management with revascularization of the celiac trunk and endovascular embolization alone. Revascularization traditionally aims to restore celiac trunk flow to prevent splanchnic ischemia by addressing the underlying cause. However, this approach may be overly dogmatic. Recent evidence suggests that revascularization might not be necessary, as embolization alone can effectively exclude the aneurysm without increasing the risk of ischemic complications thanks to multiple collateral pathways supplying the splanchnic territory. These findings challenge the need for surgical intervention in all cases and underscore the potential effectiveness of embolization alone [11,12].

The present study aims to evaluate the effectiveness and safety of retrograde embolization of high-flow PDA aneurysms without celiac trunk revascularization.

## 2. Materials and Methods

### 2.1. Study Design and Population

The local institutional review board approved this bicentric retrospective observational study (CRM 2410-423; 12 October 2024). Because of its retrospective design, written informed consent was waived.

From January 2015 to July 2024, all adult patients with high-flow PDA aneurysms secondary to celiac trunk occlusion or significant stenosis (>50%), treated exclusively by image-guided trans-arterial embolization, were included. The interventions were performed either in an emergency context or in a scheduled manner without clinical symptoms.

Non-inclusion criteria were patients with false aneurysms of PDA and the absence of significant celiac trunk stenosis on computed tomography (CT).

### 2.2. Pre Interventional Imaging

We verified the absence of any known allergy to the contrast agent before performing the CT scans. Renal function was not systematically assessed for scans conducted in emergency settings to investigate suspected bleeding, given a favorable benefit–risk balance for administering contrast. For other patients, a glomerular filtration rate (GFR) was requested for individuals over 60 and those with diabetes. If the GFR was below 30 mL/min/1.73 m^2^, an MR angiography was performed after Dotarem intravenous injection (Guerbet^®^, Villepinte, France) instead of a CT scan.

**Figure 1:**
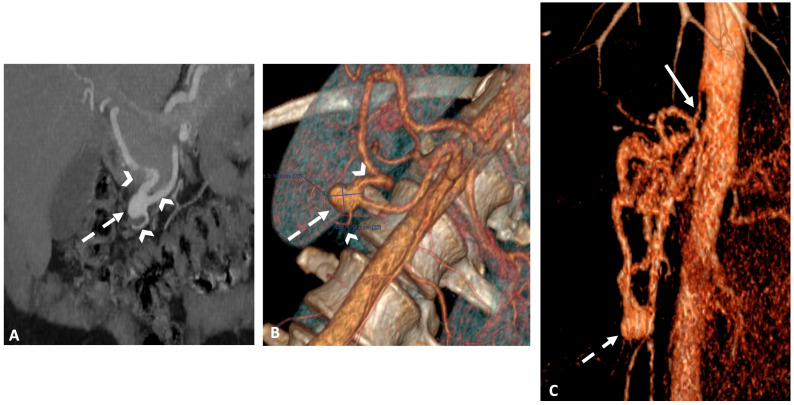
Abdominal CT after contrast injection at the arterial phase in a coronal view with maximum intensity projection reformatting (**A**), oblique 3D volume rendering (**B**), and sagittal 3D volume rendering (**C**) reconstructions showing a tight stenosis at the origin of the celiac trunk due to compression by the median arcuate ligament (solid arrow, **C**) and an unruptured aneurysm of the pancreaticoduodenal arcade (dashed arrow, (**A**–**C**)), as well as aneurysm’s inflow and outflow tracts (arrowhead, (**A**,**B**)).

All patients underwent an abdominal dynamic contrast-enhanced CT scan using a multi-detector row CT (MDCT) (Revolution CT 256; GE Healthcare, Waukesha—WI, USA or Somatom Definition AS + 128; Siemens Healthineers, Erlangen—Germany). The imaging protocol included unenhanced, arterial, and portal phase acquisitions following the intravenous administration of 1.5 mL/kg of iodinated contrast agent (Iomeron, 350-Iomeprol; Bracco Imaging, Milan, Italy) at a rate of 3–4 mL/s. A bolus-tracking technique with automated scan triggering was used, placing an elliptic region of interest in the descending thoracic aorta at the diaphragm level, with an enhancement threshold set at 100 HU. CT acquisition and reconstruction settings included a tube current range of 150–650 mA (mean: 455), a tube voltage of 120 kVp, a rotation time of 0.7 s, and a pitch of 1.375. Automatic exposure control was managed using Auto mA-Smart mA with a noise index of 25, and the field of view was set to a large body. Using model-based iterative reconstruction, images were reconstructed with a standard reconstruction kernel at a section thickness of 0.625 mm. The reconstructed images were archived in the institutional picture archiving and communication system (PACS) (Figure 1).

### 2.3. Embolization Interventions

All interventions were performed in three angiographic suites with a flat panel detector C-arm angiographic system (Allura Clarity^®^/Azurion Clarity^®^; Phillips Healthcare, Best, The Netherlands). All operators were senior staff with at least three years of experience in interventional radiology.

Patients were placed in a supine position on the angiographic table. All procedures were performed under local anesthesia using either common femoral or brachial endovascular access, with the insertion of a long 5F sheath of 55 or 90 cm, depending on the access (Flexor^®^, Cook Medical, Bloomington, IN, USA). Vascular access was through the right common femoral artery with the insertion of a 55 cm long 5F sheath in 22 patients (95.5%). In one patient, the left brachial artery was approached using a 90 cm long 5F sheath because of severe bilateral iliac stenosis.

In the case of challenging catheterization, the sheath was stabilized in the superior mesenteric artery (SMA) using the anchoring technique with a 0.014″ stiff guidewire (Spartacore^®^, Abbot, Lake County, IL, USA) in a non-target branch of the SMA [13].

All embolization interventions were performed using a retrograde approach via the SMA.

Catheterization was performed using 5F catheters, USL2 (Cordis, Miami Lakes, FL, USA)/COBRA 2 (Terumo Europe, Leuven, Belgium), and 4F catheters, BER (Cordis, Miami Lakes, FL, USA), along with 2.7F, 2.4F, and 2.0 F Progreat^®^ microcatheters (Terumo Corporation, Tokyo, Japan).

Controlled and/or uncontrolled release microcoils (0.018″) were used through two main techniques: the sandwich technique and the packing of the aneurysm sac. The sandwich technique involves embolization of the inflow and the outflow vessels of the aneurysm to completely exclude it from circulation, preventing side-branch blood flow back into the aneurysm. In the packing technique, coils are densely inserted into the aneurysm sac to fill it, thereby promoting thrombosis and excluding blood flow.

Liquid agents, cyanoacrylate glue (n-butyl cyanoacrylate metacryloxysulfolane, Glubran 2^®^; GEM, Viareggio, Italy), and Onyx^®^ 18 (LES, Covidien, Plymouth, MN, USA) were used in a critic salvage setting of hemorrhagic shock with disseminated intravascular coagulopathy, allowing fast hemodynamic status control.

Procedures were conducted under fluoroscopic or hybrid guidance using fusion between cone beam CT and fluoroscopy. Cone beam CT was used when fluoroscopy was insufficient to analyze vasculature anatomy and to guide catheterization in complex cases. The cone beam CT protocol involved a single rotation acquisition (C-arm at a head position with breath holding) following a selective injection of 30 mL of iodinated contrast agent into the SMA at a rate of 3 mL/s. Cone beam CT acquisition was performed after a 5 s delay with a tube rotation time of 5 s. Images were automatically transferred to a dedicated 3D workstation (XtraVision Release 8^®^; Philips Healthcare, Best, The Netherlands).

A final digital subtracted angiogram was performed in all patients to ensure the exclusion of the aneurysm and the retrograde injection of the splanchnic territory via collateral arteries.

For all patients, no endovascular or surgical revascularization of the celiac trunk was attempted.

### 2.4. Outcomes and Follow-Up Assessment

According to the Interventional Radiology Standards Board Guidelines [14], technical success was defined as the complete exclusion of the aneurysm on final angiographic control. Clinical success refers to the 30-day clinical outcomes assessed through clinical and imaging data. It was defined as the resolution of the signs or symptoms that initially prompted the embolization procedure, the absence of significant symptom development in initially asymptomatic patients, and the exclusion of the aneurysm on follow-up imaging.

All patients were monitored consistently across both institutions. Clinical follow up was performed by an interventional radiologist and a vascular surgeon in the unit where patients were hospitalized. Imaging follow up was conducted one month after embolization using either a 1.5T or 3T MRI system (Avanto^®^ and Skyra^®^; Siemens Healthcare, Erlangen, Germany, and Signa™ Artist; GE Healthcare, Waukesha, WI, USA) or an MDCT scanner. The imaging protocol primarily included MR angiography with intravenous contrast injection (Dotarem^®^, Guerbet, Villepinte, France).

Safety was assessed by monitoring both minor and major complications [14]. Minor complications were defined as those that did not require additional therapy and did not impact the length of hospital stay (including asymptomatic non-target embolization). Major complications were identified by the need for escalated care, such as hospital admission or prolonged hospitalization for more than 24 h, transfer from a regular floor to the intensive care unit (ICU), or complications requiring surgical intervention.

### 2.5. Statistical Analysis

The Kolmogorov–Smirnov test was used to assess the normality of quantitative variables. Continuous variables were presented as means ± standard deviations (SDs), ranges in cases of normal distribution, and as median, Q1 (25%), and Q3 (75%) in other cases. Categorical variables are reported as frequencies.

All statistical analyses were conducted using Microsoft Excel 365 (Microsoft Corporation, Redmond, WA, USA) and SPSS Version 25.0 (SPSS, Inc., Chicago, IL, USA).

## 3. Results

### 3.1. Demographics and Clinical Data

Over the study period, 23 patients met the inclusion criteria (mean age of 65 years ± 14 years [34–87 years], 12 males).

The embolization intervention was performed in an emergency setting in 12 patients (52%). Of these, ten (43%) were treated for hemorrhagic complications after aneurysm rupture and two (9%) for acute abdominal pain. The 11 remaining patients (48%) underwent elective procedures. Nine of these patients (39%) were asymptomatic, with aneurysms discovered incidentally during imaging, and the remaining two patients (9%) presented with abdominal discomfort.

An average of 1.4 aneurysms per patient ±0.65 [1,2,3] was detected on pre-embolization imaging, with a mean aneurysm size of 16 mm ± 9 mm [4–45 mm].

All patients had celiac stenosis/occlusion caused by the median arcuate ligament.

All clinical and demographic data are reported in Table 1.

### 3.2. Procedure Details

**Figure 2:**
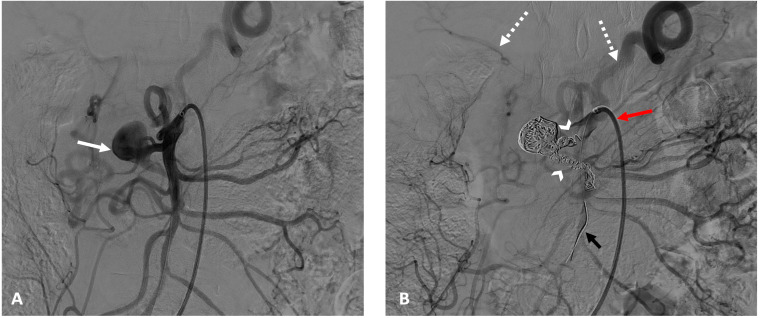
Superior mesenteric angiography at the beginning of the embolization procedure (**A**) shows an aneurysm in the gastro-duodenal arcade (solid white arrow). After embolization (**B**), a “sandwich” technique was used to exclude the entry and exit points (arrowheads) as well as the aneurysmal sac, with reinjection into splanchnic territory (dashed white arrows) via other collaterals. Note the use of a 5Fr long sheath (red arrow) and anchoring with a stiff 0.014” guidewire (black arrow) in a non-target branch for better stabilization.

In 10 cases (43%), the anchoring technique in another non-target branch of the SMA was used, enabling stability for long sheath, catheter, microcatheter, and guidewire placements and overcoming tension in the coaxial equipment (Figure 2).

The procedures were conducted under fluoroscopic guidance in eighteen patients (78%) and under hybrid guidance (fluoroscopy-cone beam CT image fusion) in the remaining five patients (22%).

Microcoils were used as the principal embolization agent in 21 patients (91%). Nineteen were treated using the sandwich technique and two had aneurysmal sac packing. Liquid agents were utilized in two cases of extreme hemorrhagic shock (Glubran 2^®^ in one patient and Onyx^®^18 in the other).

### 3.3. Technical and Clinical Success

The technical success rate was 100%, and all embolized aneurysms were excluded entirely from the final angiography.

**Figure 3:**
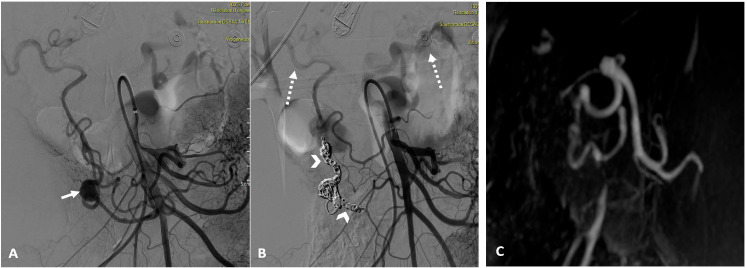
Superior mesenteric arteriography showing an aneurysm of the antero-inferior PDA before (solid white arrow/(**A**)) and after embolization (**B**) using the “sandwich” technique and exclusion of inflow and outflow arteries (arrowheads/(**B**)). Note the reinjection via collaterals into splenic and hepatic arteries (dashed arrows). (**C**) An MRI angiography in the arterial phase with subtraction in a coronal view with maximum intensity projection reformatting, confirming the total exclusion of the aneurysmal sac 1 month after embolization.

Patients were followed for a median of 16 months [2–48 months]. The clinical success rate was 100%. At the one-month follow up, imaging confirmed the complete exclusion of the embolized aneurysm in all patients (Figure 3).

During follow up, all patients showed no signs of hemorrhagic recurrence (in cases with an initial rupture), experienced resolution of abdominal pain, and had no new symptoms.

### 3.4. Embolization Safety

Three complications (14.2%) were documented, including two minor (9.5%) and one major (5%), and they were all secondary to the embolization agent’s migration into a non-target artery within the splanchnic territory.

The two minor complications were due to the migration of microcoils, one into the splenic artery in one patient and the other into the common hepatic artery in another patient. No splenic infarction or hepatic dysfunction was noted during clinical follow up or on imaging with complete arterial patency. Both patients remained utterly asymptomatic. One major complication occurred in a patient who underwent glue embolization for a ruptured aneurysm in a salvage context from severe hemorrhagic shock. This non-target embolization was complicated by a total splenic infarction, leading to infection and necessitating admission to the intensive care unit and a splenectomy.

Technical details, outcomes, and follow-up data are reported in Table 2.

## 4. Discussion

This study suggests that the embolization of PDA aneurysms via a retrograde SMA approach, without celiac trunk revascularization, is an effective and safe treatment modality.

Endovascular techniques offer numerous advantages over traditional surgical treatment of aneurysms with celiac trunk revascularization, including reduced morbidity, a shorter recovery time, and lower complication rates. These benefits make the endovascular approach preferable for most patients, as evidenced in our study and supported by the existing literature [1,15,16,17].

The present study’s data demonstrate a 100% technical success rate, with complete aneurysm exclusion confirmed on final angiographic control and one-month follow-up imaging. The clinical success rate was also 100%, with all patients experiencing resolution of symptoms or remaining asymptomatic. These outcomes align with previous studies, which report similarly high success rates for endovascular interventions in PDA aneurysms [18,19,20,21]. Achieving these outcomes requires meticulous anatomical study prior to embolization and careful procedural planning. High-resolution imaging modalities, such as dynamic CT with 3D reconstruction, are crucial for pre-procedural planning. These modalities allow for precise mapping of vascular anatomy and facilitate safe and effective interventions [1].

Despite the high success rates, specific technical challenges must be addressed, particularly during catheterization in ruptured aneurysms requiring rapid management. In the present study, the use of a long 5F sheath in all patients and the relatively frequent application of the anchoring technique (43% of patients) effectively mitigated these challenges. This approach provided better stability during the procedure, ensuring that catheters, microcatheters, and guidewires remained securely positioned, even in the most challenging vascular anatomies [13]. Additionally, cone beam CT, used in 22% of our patients, not only aids in better planning and endovascular navigation but also allows for a more detailed anatomical study. This guidance modality significantly improves the accuracy and safety of these procedures, which is crucial for successful embolization in complex cases [22,23].

The low incidence of ischemic complications underscores the safety of endovascular embolization via the retrograde SMA approach. This low complication rate observed in the present study aligns with the literature, confirming the safety of this technique when properly implemented [18,19,20]. The safety of the endovascular approach is attributed to the extensive collateral network in the splanchnic circulation, including multiple pancreaticoduodenal arcades that can compensate for the occlusion of the primary arteries. These collateral pathways, observed in 87% of our patients on final angiography, ensure adequate perfusion even after embolization, thereby mitigating the risk of ischemic damage [24,25,26].

In the present cohort, the only patient who had an ischemic complication of the spleen was initially in severe hemorrhagic shock and was embolized with glue. This ischemic complication can be mainly attributed to the non-target migration of glue into the splenic artery and partly to vasoconstriction in the splanchnic territory secondary to shock, which makes supplementation via the collaterals less effective.

This study has some limitations that need to be acknowledged. First, its retrospective design with a relatively small patient sample may limit the robustness of our results. However, this should be interpreted in context, as this is a rare pathology. To our knowledge, the present series represents the largest published on endovascular embolization of PDA aneurysms via a retrograde SMA approach. Moreover, the absence of a control group in which celiac trunk recanalization was performed limits the robustness of our results. However, the median clinical and radiological follow up of 16 months may ensure the effectiveness and the safety of the approach based uniquely on the aneurysms’ embolization, without celiac trunk recanalization. Moreover, to minimize potential multicenter bias, we ensured consistency between the two centers regarding equipment and procedural protocols. Both centers used high-quality imaging systems and comparable embolization materials, pre-interventional imaging, and embolization techniques. This consistency helped to minimize variability, allowing for reliable comparison of outcomes and ensuring that center-specific factors did not influence the differences in the results.

## 5. Conclusions

Endovascular embolization of high-flow PDA aneurysms via a retrograde SMA approach is sufficient, effective, and safe, even without complementary recanalization of the celiac trunk. This technique can be proposed as the first-line approach for managing these high-risk bleeding aneurysms. It should be scheduled in nearly asymptomatic patients to prevent aneurysm rupture independently of their size. Meanwhile, accurate intervention planning based on high-quality pre-interventional imaging and appropriate embolization techniques is crucial to ensuring technical and clinical success.

## Figures and Tables

**Table 1:** Demographics and clinical data of included patients with high-flow PDA.

Patient	Sex	Age (y)	Circumstances of Discovery	Urgent/Scheduled Embolization	Size of PDA (mm)	Celiac Trunk Occlusion or Stenosis (>50%)
1	F	87	Rupture	Urgent	16	Yes
2	M	48	Fortuitous	Scheduled	20	Yes
3	M	64	Rupture	Urgent	4	Yes
4	F	58	Rupture	Urgent	12	Yes
5	F	53	Rupture	Urgent	15	Yes
6	M	79	Fortuitous	Scheduled	17	Yes
7	M	49	Fortuitous	Scheduled	13	Yes
8	M	63	Rupture	Urgent	15	Yes
9	F	47	Abdominal discomfort	Scheduled	15	Yes
10	F	60	Fortuitous	Scheduled	19	Yes
11	M	53	Abdominal discomfort	Scheduled	15	Yes
12	M	64	Rupture	Urgent	26	Yes
13	M	85	Acute abdominal pain	Urgent	8	Yes
14	F	78	Fortuitous	Scheduled	17	Yes
15	M	71	Acute abdominal pain	Urgent	4	Yes
16	F	47	Rupture	Urgent	34	Yes
17	F	34	Fortuitous	Scheduled	17	Yes
18	F	80	Rupture	Urgent	5	Yes
19	F	68	Fortuitous	Scheduled	9	Yes
20	F	76	Fortuitous	Scheduled	45	Yes
21	M	67	Fortuitous	Scheduled	15	Yes
22	M	83	Rupture	Urgent	9	Yes
23	M	77	Rupture	Urgent	18	Yes

PDA: pancreaticoduodenal arcade aneurysm; M: male; F: female.

**Table 2:** Embolization technical details, follow up, and outcome data.

Patient	Embolization Agent	Embolization Technique	Reinjection of Splanchnic Territory on Final Arteriogram	Technical Success	Complications	Duration of Follow Up (Months)	Exclusion of Aneurysm on 1-Month Imaging
1	Microcoils	Sandwich	Yes	Yes	No	14	Yes
2	Microcoils	Packing	Yes	Yes	No	73	Yes
3	Microcoils	Sandwich	No HA	Yes	No	51	Yes
4	Microcoils	Sandwich	Yes	Yes	No	37	Yes
5	Microcoils	Sandwich	Yes	Yes	No	16	Yes
6	Microcoils	Sandwich	Yes	Yes	No	18	Yes
7	Microcoils	Sandwich	Yes	Yes	No	75	Yes
8	Microcoils	Packing	Yes	Yes	No	65	Yes
9	Microcoils	Sandwich	Yes	Yes	No	37	Yes
10	Microcoils	Sandwich	Yes	Yes	No	48	Yes
11	Microcoils	Sandwich	Yes	Yes	No	1	Yes
12	Glubran 2^®^	Occlusion	No HA + SA	Yes	Glue migration into the SA and HA	1	Yes
13	Microcoils	Sandwich	Yes	Yes	No	61	Yes
14	Microcoils	Sandwich	Yes	Yes	No	1	Yes
15	Microcoils	Sandwich	Yes	Yes	No	2	Yes
16	Microcoils	Sandwich	Yes	Yes	No	2	Yes
17	Microcoils	Sandwich	Yes	Yes	No	7	Yes
18	Onyx^®^18	Occlusion	Yes	Yes	No	27	Yes
19	Microcoils	Sandwich	Yes	Yes	No	20	Yes
20	Microcoils	Sandwich	No SA	Yes	Coil migration into the HA	5	Yes
21	Microcoils	Sandwich	Yes	Yes	No	9	Yes
22	Microcoils	Sandwich	Yes	Yes	Coil migration into the SA	3	Yes
23	Microcoils	Sandwich	Yes	Yes	No	1	Yes

HA: hepatic artery; SA: splenic artery.

## Data Availability

The datasets used and/or analyzed during the current study are available from the corresponding author upon reasonable request.
